# Comorbidity and Treatment in Older Psychiatric In-patients—A Retrospective Study in a Chinese Psychiatric Hospital

**DOI:** 10.3389/fpsyt.2021.722329

**Published:** 2021-10-26

**Authors:** Hongmei Liu, Yuncheng Zhu, Xiaohui Wu, Kan He, Xiaoxiao Wang, Ping Sun, Jie Zhao, Yamin Yao, Juanjuan Ren, Ruizhi Mao, Tao Yang, Lu Yang, Xiujia Sun, Ping Jiang, Chen Zhang, Yiru Fang

**Affiliations:** ^1^Clinical Research Center, Shanghai Mental Health Center, Shanghai Jiao Tong University School of Medicine, Shanghai, China; ^2^Shanghai Key Laboratory of Psychotic Disorders, Shanghai, China; ^3^Laboratory of Biochemistry and Pharmacology, Shanghai Mental Health Center, Shanghai Jiao Tong University School of Medicine, Shanghai, China; ^4^Division of Mood Disorders, Shanghai Hongkou Mental Health Center, Shanghai, China; ^5^Department of Bioinformatics and Biostatistics, School of Life Sciences and Biotechnology, Shanghai Jiao Tong University, Shanghai-Yale Joint Center for Biostatistics and Data Science, Shanghai Jiao Tong University, Shanghai, China; ^6^Qingdao Mental Health Center, Qingdao University, Qingdao, China; ^7^Department of Anatomy and Histology and Embryology, Kunming Medical University, Kunming, China; ^8^Center for Excellence in Brain Science and Intelligence Technology, Academy of Sciences of China, Shanghai, China

**Keywords:** older psychiatric patients, somatic comorbidity, combined drug treatment, polypharmacy, mental illness

## Abstract

**Background:** Comorbid somatic diseases increase the death risk and affect the condition, treatment, and prognosis of older psychiatric patients. We investigated the comorbidity and drug treatment in older patients with psychosis.

**Methods:** This retrospective study used data from 3,115 older psychiatric in-patients hospitalized at the Shanghai Mental Health Center Affiliated to Shanghai Jiaotong University School of Medicine, China discharged from 2005 to 2015. Descriptive analyses of patients' age, sex, treatment drugs, diagnoses (based on ICD-10), and time trend were performed.

**Results:** Patients' median age was 56 (range, 50-98) years; 1,824 (58.6%) were female. The top five first-level diagnoses were schizophrenia (F20) (*n* = 1,818, 58.3%), depressive episode (F32) (*n* = 457, 14.6%), bipolar affective disorder (F31) (*n* = 151, 4.8%), manic episode (F30), (*n* = 143, 4.6%), and vascular dementia (F01) (*n* = 136, 4.4%). Mental (99.9%), central nervous system (85.2%), digestive system (83.5%), cardiovascular system (72.5%), and anti-infective (59.6%) drugs had the highest prescription rates. The combined use of antidepressants, anti-anxiety, anti-arrhythmic, hormones and endocrine system drugs were significantly higher in female than in male patients, while mood stabilizers and genitourinary system drugs significantly more frequent in men. With increasing age, the F20-F29 patients decreased, while F00-F09 patients increased, with the corresponding changes to prescription in those patients. In comparison to that in 2005-2010, the combined prescriptions for genitourinary and cardiovascular drugs increased between 2011 and 2015, and F00-F09 and F40-F48 older patients doubled, accordingly anti-Alzheimer's disease drugs and antidepressants more than doubled. F30-F39 patients increased by 49.1%, and anti-anxiety drugs, mood stabilizers, etc. increased by ≥50%; F20-F29 older patients decreased by 26.7%, while antipsychotics only increased by 4.4%.

**Conclusions:** This study found the combined drug treatment of somatic diseases, particularly for central nervous, digestive, cardiovascular, respiratory and genitourinary drugs were extremely common among older psychiatric in-patients in China. With increasing age, the F20-F29 patients decreased, while F00-F09 patients increased; the antipsychotics prescriptions decreased, and almost all comorbidity drugs increased. Compared with that in 2005-2010, the older patients with all diagnosis except F20-F29 increased in 2011-2015, and the prescriptions for psychotropic, genitourinary, and cardiovascular drugs increased.

## Introduction

The worldwide population is aging (1). A direct consequence of an aging population and increasing social pressure is a surge in the number of older mental patients (2). Studies have shown that about 2% of older people suffer from severe mental illness (3). A cross-sectional epidemiological survey from China showed that the weighted 12-month prevalence of mental disorders in patients >65 years old was 4.0% (2.8-5.2%) (4). Another semi-structured interview study from Nepal (5) showed that about 18% of patients >60 years of age may have a diagnosable mental illness, suggesting that older patients are more likely to suffer from diagnosable mental illness.

Older patients with mental disorders, especially severe mental diseases, suffer from more physical and cognitive impairment and social function disability (6), which further damages their physical health. Compared with regular older patients, the risk of somatic comorbidity with mental illness doubles, and the risk of multiple (≥2) somatic diseases increases significantly (odds ratio = 4.1) (7). In fact, about 30-64% of older psychiatric patients suffer from ≥1 physical diseases (7–10). As many as 75% of older mental patients re-admitted to hospital suffer from physical comorbidities (11), particularly those affecting the cardiovascular, respiratory, nervous, endocrine, and digestive systems (11, 12), which may be related to an unhealthy lifestyle and diet (e.g., excessive smoking, insufficient dietary intake, and irregular diet) (13–15), side effects of psychotropic drugs (16), metabolic disorders (17), physical activity limitations, and lack of physical exercise (7, 17).

Physical comorbidities increase the risk of death in older patients with psychosis (18); a previous study reported that the mortality rate in older patients with psychosis was almost tripled than that of older persons with mental health (18.0 vs. 9.7%) (19). Simultaneously, physical comorbidities could also affect the severity of mental illness symptoms (20), increase the emergency admission rate, prolong the hospital stay, and increase the number of rehospitalizations (19, 21). Therefore, rapid and effective treatment of the physical comorbidities may greatly benefit the treatment and prognosis of mental illness. However, as attention may focus only on the mental illness, older patients with mental illness may not seek treatment for physical complications (13, 14, 16), or the necessary physical examination could be lacking (22); in such cases, the physical comorbidities may not be found (23–25), have a delayed diagnosis (11), or be improperly treated (23, 26–28). In general hospitals, >52% of older patients took hypnotics or psychotropic drugs (29) or received ≥1 psychotropic drug treatment (30) during hospitalization. As many as 84.8% of the in-patients in psychiatric hospitals with severe mental illness have somatic comorbidities, of which 77.7% use drugs for the treatment of somatic comorbidities, and 67.7% multi-drug combination therapy (31).

In many countries, psychiatric and physical diseases are often treated in different hospitals, and doctors often not allowed to access the diagnosis and treatment records of other hospitals due to patient privacy, data protection, and medical system isolation (32). As a result, psychiatric hospitals are often unable to assign a comprehensive and professional diagnosis of physical diseases. In the actual process of diagnosis and treatment, doctors in psychiatric hospitals generally rely on patients or their relatives to provide the diagnosis and treatment records from the general hospital. Therefore, this study aimed to retrospectively analyze relevant data on prescription use of psychotropic drugs and somatic disease treatment drugs in older psychiatric patients to indirectly study the somatic complications and drug treatment of older psychiatric patients, thereby providing new clues for the treatment of somatic complications in older patients with mental illness.

## Materials and Methods

### Study Design and Database

This retrospective, observational, single-center, cross-sectional study was performed at the Shanghai Mental Health Center Affiliated to Shanghai Jiaotong University School of Medicine (China), the second largest psychiatric hospital in China. Data were extracted from the electronic prescription and administration databases. For this study, available drug prescription charts of older in-patients with mental illness, aged ≥50 years, discharged from the hospital between January 2005 and October 2015 were analyzed, without any limit in the diagnosis and medication. The database also listed all medications administered to the patient by the medical staff during their stay. All data related to personal identification information, such as name and hospitalized identification number, were anonymized and encoded as data files. If a patient was hospitalized twice or more during the study period, they were not counted as a new patient provided the International Classification of Diseases (ICD-10) diagnosis remained. The study was approved by the ethics committee of Shanghai Mental Health Center.

The demographic data collected included sex, age at admission, ICD-10 psychiatric diagnosis at discharge, and number and duration of hospitalizations. For the present study, the main psychiatric diagnoses were categorized according to the ICD-10 as follows: (1) organic, including symptomatic, mental disorders (F00-F09); (2) mental and behavioral disorders due to psychoactive substance use (F10-F19); (3) schizophrenia, schizotypal, and delusional disorders (F20-F29) (4) mood [affective] disorders (F30-F39); (5) neurotic, stress-related, and somatoform disorders (F40-F48); and (6) other mental and behavioral disorders (F50-F99). We then analyzed the drug treatment of somatic diseases among the top five diagnoses with the highest number of patients, including schizophrenia (F20), depressive episode (F32), bipolar affective disorder (F31), manic episode (F30), and vascular dementia (F01). Detailed information regarding the primary, secondary, and tertiary diagnoses and subtypes of disease classification based on the ICD-10 is displayed in Supplemental Table 1.

All prescribed psychotropic drugs were administered throughout the entire hospital stay. All treatment drugs were analyzed, except those applied topically or Chinese traditional medicine, which may have an ambiguous or unknown mechanism of action. Based on the use and targeted treatment organs/systems, the therapeutic drugs were divided into 11 categories, which are defined in detail in **Table 2**. The first three drug categories (psychotropic drugs, central nervous system drugs, and cardiovascular system drugs) were subdivided based on differences in pharmacological effects (**Table 2**). As during the hospitalization period, patients may receive drugs in different categories or several subcategories within the same category, in this study, we only studied categories or subcategories of drugs without considering the specific drugs; that is, regardless whether the patient used 1 or ≥2 drugs in these category, the patient was included in this category of drugs.

### Statistical Analysis

We performed a descriptive analysis of the following parameters: age, sex, number of patients receiving a specific kind of drug, and specific category diagnosis. Categorical variables are expressed as numbers (percentages), while continuous variables are presented as medians and ranges, if not normally distributed. Changes in patient numbers were assessed using the Kruskal-Wallis test, and changes in drug frequency tested using the Chi-square test. Differences were considered statistically significant at *P* < 0.05. Analyses were conducted using SPSS version 19.0.

## Results

### Demographic Data

A total of 6,745 inpatient records were available for extraction, and 3,115 patients included in the analysis by excluding repeated hospitalization records. Of these, 1,291 (41.4%) were men, with a median age of 55 (range, 50-98) years, and 1,824 (58.6%) were women, with a median age of 56 (range, 50-98) years (z = −3.219, *P* = 0.001) (Supplemental Table 2). Table 1 shows the distribution of the number of older patients according to age, as well as their main diagnoses based on ICD-10. The most common diagnoses included F20-F29, F30-F39, and F00-F09. Patients with the above three diagnoses accounted for 95.1% (*n* = 2,962) of the total number of patients in this study. The remaining 153 patients belonged to the other diagnostic categories. Among them, significantly more (*P* < 0.0001) males than females were diagnosed with mental and behavioral disorders due to psychoactive substance use (F10-F19); however, significantly more female patients were diagnosed with mood [affective] disorders (F30-F39) than male patients (*P* = 0.048). In addition, age differences were significant among patients with different diagnoses (*P* < 0.0001); patients with F00-F09 diagnoses were significantly older than those in the other diagnosis categories (Supplemental Table 2).

**Table 1:** Characteristics of the study population.

**Characteristics**	**Frequencies**
Total patients	3,115
- Gender, *n* (%)	
- Male	1,291 (41.4)
- Female	1,824 (58.6)
- Age in years, median (range)	56 (50-98)
- Subgroups divided by age (years, yr)	n (%)
−50-59 yr	2,117 (67.9)
−60-69 yr	638 (20.5)
−70-79 yr	252 (8.1)
−80-89 yr	96 (3.1)
−90-99 yr	12 (0.4)
Diagnosis based on ICD-10, *n* (%)	n (%)
- Organic, including symptomatic, mental disorders (F00-F09)	242 (7.8)
- Mental and behavioral disorders due to psychoactive substance use (F10-F19)	34 (1.1)
- Schizophrenia, schizotypal and delusional disorders (F20-F29)	1,969 (63.2)
- Mood [affective] disorders (F30-F39)	751(24.1)
- Neurotic, stress-related and somatoform disorders (F40-F48)	83 (2.7)
- The other mental and behavioral disorders based on ICD-10 (F50-F99)	36 (1.2)

### Drug Treatment and In-patient Gender

Table 2 summarizes drug prescription types and rates in older in-patients. The prescription rate of psychotropic drugs in female patients was higher than in male patients, a difference was statistically significant for antidepressant drugs (male 36.9% vs. female 47.3%; *P* = 638e-9), mood stabilizers (male 44.0% vs. female 40.2%; *P* = 0.034), and anti-anxiety drugs (male 45.4% vs. female 51.9%; *P* = 0.0003). The combined use of antiarrhythmic drugs (male 15.3% vs. female 22.5%; *P* = 4.5208e-7) and anti-heart failure, anti-shock, and circulatory system drugs (male, 30.8% vs. female 35.4%; *P* = 0.007) was significantly higher in females than in males; however, male patients (18.5%) received significantly more genitourinary drugs than females (14.6%). In addition, combinations of hormone and endocrine system drugs (*P* = 0.006) and anti-tremor paralytic drugs (*P* = 0.001) also showed significant gender differences. There were no gender differences in the prescription rates of other therapeutic drugs, including those targeting the central nervous system, digestive tract, bone and joint complications, and respiratory system.

**Table 2:** Most frequently used drugs of older psychiatric in-patients comorbid somatic diseases in this study.

	**Total *n* (%)**	**Men *n* (%)**	**Women *n* (%)**	**χ2, *P***
1. Psychotropic drugs	3,113 (99.9)	1,290 (41.4)	1,823 (58.6)	0.000, 1.000
1.1 antipsychotics	3,093 (99.3)	1,283 (41.5)	1,810 (58.5)	0.236, 0.627
1.2 antidepressants	1,339 (43.0)	476 (35.5)	863 (64.5)	33.638, 6.638E-9^**^
1.3 mood stabilizer	1,301(46.8)	568 (43.7)	733 (56.3)	4.513, 0.034^*^
1.4 anti-anxiety drugs	1,533 (49.2)	586 (38.2)	947 (61.8)	12.888, 0.0003^**^
1.5 sedative hypnotic drugs	1,430(45.9)	547 (38.3)	883 (61.7)	11.105, 0.001
1.6 anti-Alzheimer's drugs	857 (27.5)	356 (41.5)	501 (58.5)	0.004, 0.947
2. Central nervous system drugs	2,655 (85.2)	1,115 (42.0)	1,540 (58.0)	2.254, 0.133
2.1 antiepileptics and anticonvulsants	2,124 (68.2)	905 (42.6)	1,219 (57.4)	3.725, 0.054
2.2 anti-tremor paralytic drugs	1,318 (42.3)	533 (40.4)	785 (59.6)	0.950, 0.330
2.3 brain metabolism and circulation improvers	462 (14.8)	187 (40.5)	275 (59.5)	0.210, 0.647
2.4 analgesics / antipyretics, analgesics, analgesics	390 (12.5)	179 (45.9)	211 (54.1)	3.642, 0.056
2.5 nerve center agonists, vertigo drugs and other central drugs	776 (24.9)	338 (43.6)	438 (56.4)	1.900, 0.168
3. Cardiovascular drugs	2,257 (72.5)	935 (41.4)	1322 (58.6)	0.001, 0.974
3.1 antihypertensive drugs	1,498 (48.1)	638 (42.6)	860 (57.4)	1.560, 0.212
3.2 lipid regulating drugs	283 (9.1)	107 (37.8)	176 (62.2)	1.695, 0.193
3.3 hypoglycemic drugs	781 (25.1)	327 (41.9)	454 (58.1)	0.077, 0.781
3.4 antiarrhythmic drugs	608 (19.5)	197 (32.4)	411 (67.6)	25.458, 4.521E-7^**^
3.5 antithrombotic drugs	101 (3.2)	47 (46.5)	54 (53.5)	1.114, 0.291
3.6 anti-angina and coronary heart disease drugs	442 (14.2)	188 (42.5)	254 (57.5)	0.252, 0.616
3.7 anti-heart failure, anti-shock and other drugs for improving circulatory system	1,042 (33.5)	397 (38.1)	645 (61.9)	7.218, 0.007^**^
4. Digestive tract drugs	2,602 (83.5)	1,068 (41.0)	1,534 (59.0)	1.038, 0.308
5. Genitourinary drugs	506 (16.2)	239 (47.2)	267 (52.8)	8.341, 0.004^**^
6. Drugs related to bone and joint	459 (14.7)	185 (40.3)	274 (59.7)	0.288, 0.591
7. Respiratory drugs	442 (14.2)	195 (44.1)	247 (55.9)	1.517, 0.218
8. Hormone and endocrine system drugs	391 (12.6)	137 (35.0)	254 (65.0)	7.561, 0.006^**^
9. Vitamins, minerals and other nutrients	1,546 (49.6)	604 (42.9)	882 (57.1)	2.864, 0.091
10. Anti-tumor drugs	138 (4.4)	38 (27.5)	100 (72.5)	11.510, 0.001^**^
11. Anti-infective drugs	1,857 (59.6)	783 (42.2)	1,074 (57.8)	0.983, 0.332

*The values in bold indicated that the P- value was statistically significant*.

* *P <0.05*,

** *P <0.01*.

### Drug Treatment and Psychiatric Diagnosis

We divided the patients into six different first-class diagnostic categories according to the ICD-10 classification criteria and the number of patients and analyzed the relationship between disease diagnoses and the percentage of combination medication (Supplementary Table 3). We focused on the top five first-class diagnoses with the largest number of patients (F20, F32, F31, F30, and F01) and the utilization rate of combined treatment drugs for somatic diseases (Table 3). The results showed that regardless of the diagnostic classification, psychotropic drugs, especially antipsychotics, had the highest prescription rate (Supplementary Table 3).

**Table 3:** Major prescribed drugs for somatic complications of older psychiatric in-patients related to the top five psychiatric diagnoses based on ICD-10.

	**F20 *n* (%)**	**F32 *n* (%)**	**F31 *n* (%)**	**F30 *n* (%)**	**F01 *n* (%)**
Patients number	1,818 (100.0)	457 (100.0)	151 (100.0)	143 (100.0)	136 (100.0)
Central nervous system drugs	1,600 (88.0)	377 (82.5)	129 (85.4)	119 (83.2)	115 (84.6)
- antiepileptics and anticonvulsants	1,294 (71.2)	337 (73.7)	113 (74.8)	97 (67.8)	35 (25.7)
- anti-tremor paralytic drugs	1,006 (55.3)	73 (16.0)	47 (31.1)	51 (35.7)	22 (16.2)
- brain metabolism and circulation improvers	221 (12.2)	57 (12.5)	17 (11.3)	15 (10.5)	92 (67.6)
- analgesics/antipyretics, analgesics, analgesics	249 (13.7)	43 (9.4)	9 (6.0)	17 (11.9)	25 (18.4)
- nerve center agonists, vertigo drugs and other central drugs	504 (27.7)	46 (10.1)	4.0 (26.5)	45 (31.5)	49 (36.0)
Cardiovascular drugs	1,350 (74.3)	299 (65.4)	101 (66.9)	90 (62.9)	132 (97.1)
- antihypertensive drugs	871 (47.9)	211 (46.2)	71 (47.0)	64 (44.8)	95 (69.9)
- lipid regulating drugs	152 (8.4)	26 (5.7)	11 (7.3)	12 (8.4)	50 (36.8)
- hypoglycemic drugs	471 (25.9)	83 (18.2)	30 (19.9)	30 (21.0)	70 (51.5)
- antiarrhythmic drugs	378 (20.8)	48 (10.5)	19 (12.6)	21 (14.7)	66 (48.5)
- antithrombotic drugs	51 (2.8)	7 (1.5)	8 (5.3)	2 (1.4)	12 (8.8)
- anti-angina and coronary heart disease drugs	219 (12.0)	52 (11.4)	23 (15.2)	20 (14.0)	65 (47.8)
- anti-heart failure drugs, anti-shock drugs, procoagulant drugs, improving circulation activation, etc	581 (32.0)	124 (27.1)	47 (31.1)	40 (28.0)	108 (79.4)
Digestive tract drugs	1,539 (84.7)	368 (80.5)	111 (73.5)	123 (86.0)	133 (97.8)
Genitourinary drugs	225 (12.4)	54 (11.8)	23 (15.2)	24 (16.8)	92 (67.6)
Drugs related to bone and joint	328 (18.0)	48 (10.5)	20 (13.2)	14 (9.8)	12 (8.8)
Respiratory drugs	250 (13.8)	23 (5.0)	15 (9.9)	13 (9.1)	81 (59.6)
Hormone and endocrine system drugs	188 (10.3)	45 (9.8)	17 (11.3)	14 (9.8)	65 (47.8)
Vitamins, minerals and other nutrients	884 (48.6)	188 (41.1)	68 (45.0)	70 (49.0)	121 (89.0)
Anti-tumor drugs	88 (4.8)	15 (3.3)	6 (4.0)	3 (2.1)	13 (9.6)
Anti-infective Drugs	1,133 (62.3)	211 (46.2)	81 (53.6)	80 (55.9)	123 (90.4)

*F20, Schizophrenia; F32, Depressive episode; F31, Bipolar affective disorder; F30, Manic episode; F01, Vascular dementia; the data in this table were presented as the number and percentage of patients, patients number (%)*.

In patients diagnosed with mood [affective] disorders (F30-F39), the combined use of antidepressants (72.6%) and mood stabilizers (57.3%) was the most common. It is worth noting that even if they were in the same diagnosis in the scope of F30-F39, the combined use of somatic disease treatment drugs would change due to the different primary diagnoses of the patient. To be exact, in addition to the above two drug categories (antidepressants and mood stabilizers), anti-inflammatory drugs (72.2%) were most common in patients with a depressive episode (F32), and antiepileptic and anticonvulsant drugs (74.8%) were most common in patients with bipolar affective disorder (F31). However, compared with several other first-level diagnoses, the combination of cardiovascular drugs (62.9%) and antidepressants (27.3%) were seldom used in patients with manic epochs (F30). Patients with bipolar affective disorder (F31) had the least combined use of digestive tract drugs (73.5%), antipyretic and analgesic drugs (6.0%), and anti-Alzheimer's drugs (16.6%). In addition, the combination use rate of almost all subcategories of cardiovascular drugs, hormones, and endocrine system drugs were lowest in patients with depressive episode (F32) (Table 3 and Supplementary Table 3).

Analysis of the combined use of a variety of specific types of physical disease treatment drugs showed that compared with other diagnoses, cardiovascular drugs (97.1%), digestive tract drugs (97.8%), genitourinary drugs (67.6%), and respiratory drugs (59.6%) were the most commonly used in combination with vascular dementia (F01) patients, while central nervous system drugs (88.0%) and drugs related to bones and joints (18.0%) were most commonly used in patients with schizophrenia (F20). Central nervous system drugs (82.5%), genitourinary drugs (11.8%), and respiratory drugs (5.0%) had the lowest combined use rate for patients with depressive episodes (F32). In addition, there were three cases with the least use of drugs for physical diseases, and the drug classification and first-class diagnosis of mental diseases were as follows: cardiovascular drugs and manic episodes (F30) (62.9%), digestive tract drugs and bipolar effective disorder (F31) (73.5%), and drugs related to bone and joint and vascular dementia (F01) (8%).

### Time Trend of Drug Prescription

**Figure 1:**
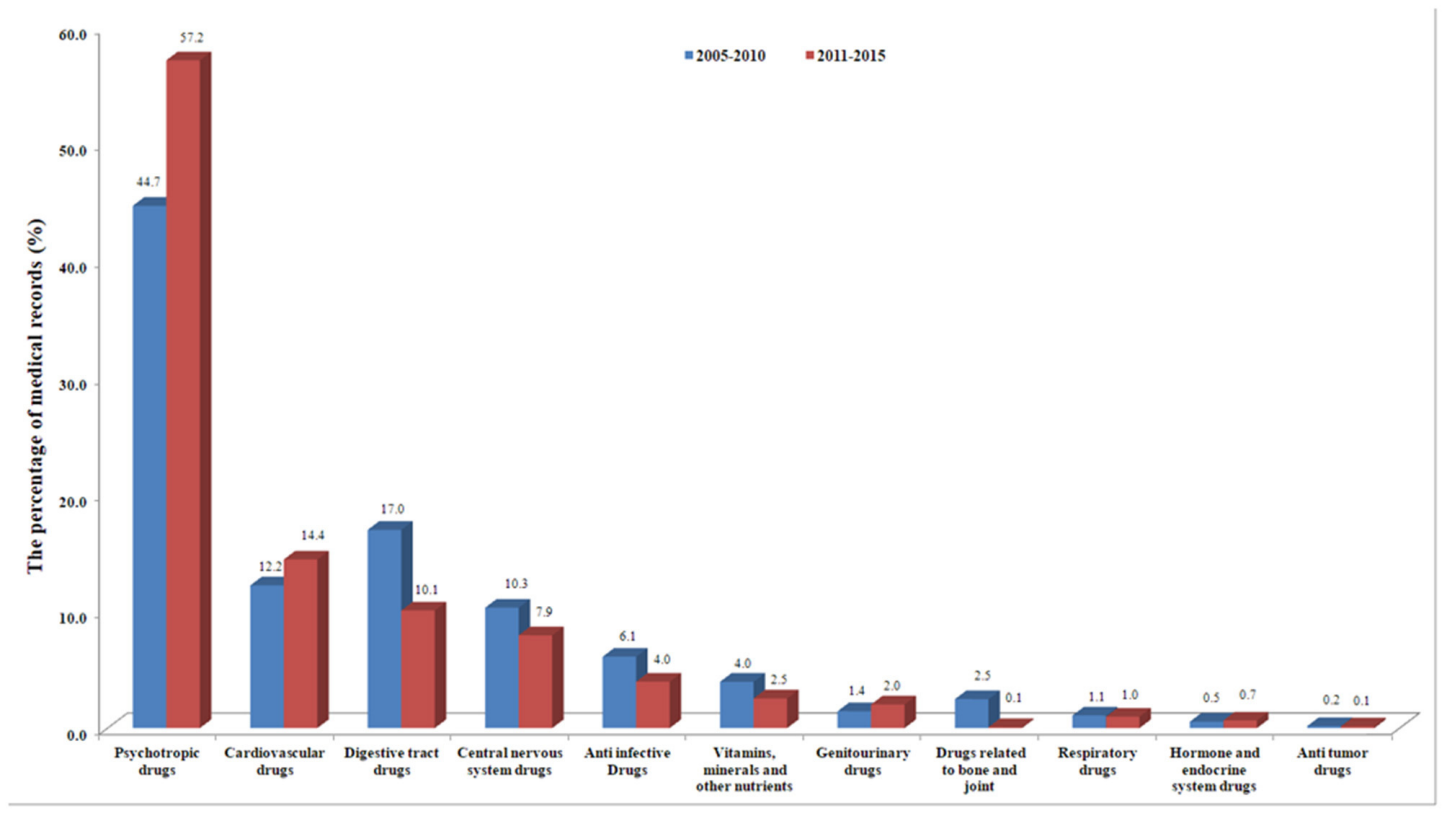
Time trend of drug prescriptions in two time periods of the medical records.

In this study, a total of 442,145 prescription records were originally obtained, and 293,099 records were available for analysis by excluding traditional Chinese medicine and drugs applied topically. In order to analyze the time trend of drug prescription and psychiatric diagnosis, we divided the time covered in this study into two periods: (1) 2005-2010*;* (2) 2011-2015. Descriptive analysis was performed as a percentage of target drug prescription records among the total number of prescriptions (or the number of target diagnostic patients in the total patients). The results showed that, compared with that in 2005-2010, the percentage of patients diagnosed with F00-F09 (increased from 13.8 to 28.8%) and F40-F48 (from 2.1 to 4.8%) among older psychiatric patients in 2011-2015 more than doubled. The percentage of older patients with F10-F19 (from 0.5 to 0.8%) and F30-F39 (from 16.1 to 24.0%) increased by 60.0 and 49.1%, respectively, while the percentage of older patients with F20-F29 decreased from 66.8 to 40.1% (Figure 1 and Supplementary Table 4).

**Figure 2:**
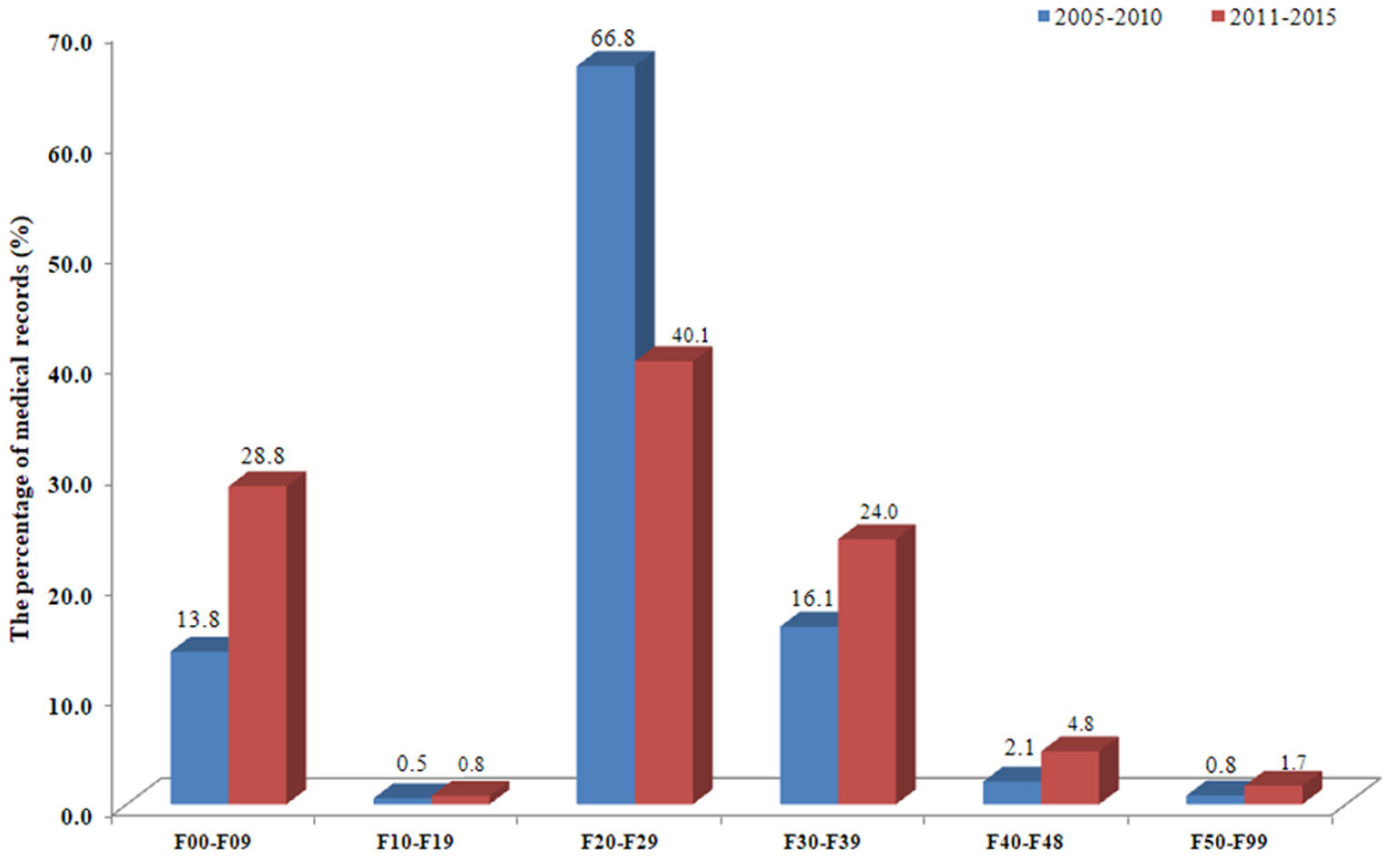
Time trends of major psychiatric diagnoses in two time periods of the medical records.

Regarding psychotropic drug prescriptions in medical records, compared with 2005-2010, the number of psychotropic drug prescriptions in 2011-2015 increased from 44.7 to 57.2%. Among them, antidepressants and anti-Alzheimer's drugs increased the most, both doubling or more, the former increased from 4.1 to 8.2%, the latter from 2.1 to 4.5%; followed by anti-anxiety drugs, which increased from 3.1 to 5.6%; mood stabilizers increased from 3.0 to 4.5%, and sedative hypnotics increased from 2.3 to 3.4%. Antipsychotics (from 29.8 to 31.1%) increased the least, only 4.4%. Among the drugs for the treatment of comorbid somatic diseases, genitourinary drugs increased from 1.4 to 2.0%. Cardiovascular drugs increased from 12.2 to 14.4%, among which, antihypertensive drugs increased by 47.4% (from 3.8 to 5.6%), as did other cardiovascular drugs. While digestive tract drugs decreased from 17.0 to 10.1%; central nervous system drugs from 10.3 to 7.9%; anti-infectious drugs from 6.1 to 4.0%; and drugs related to bone and joint from 2.5 to 1.0%. In addition, the number of prescription records of respiratory drugs, hormone and endocrine system drugs and anti-tumoral drugs decreased to varying degrees (Figure 2 and Supplementary Table 5).

### Age Trend of Drug Prescription

**Figure 3:**
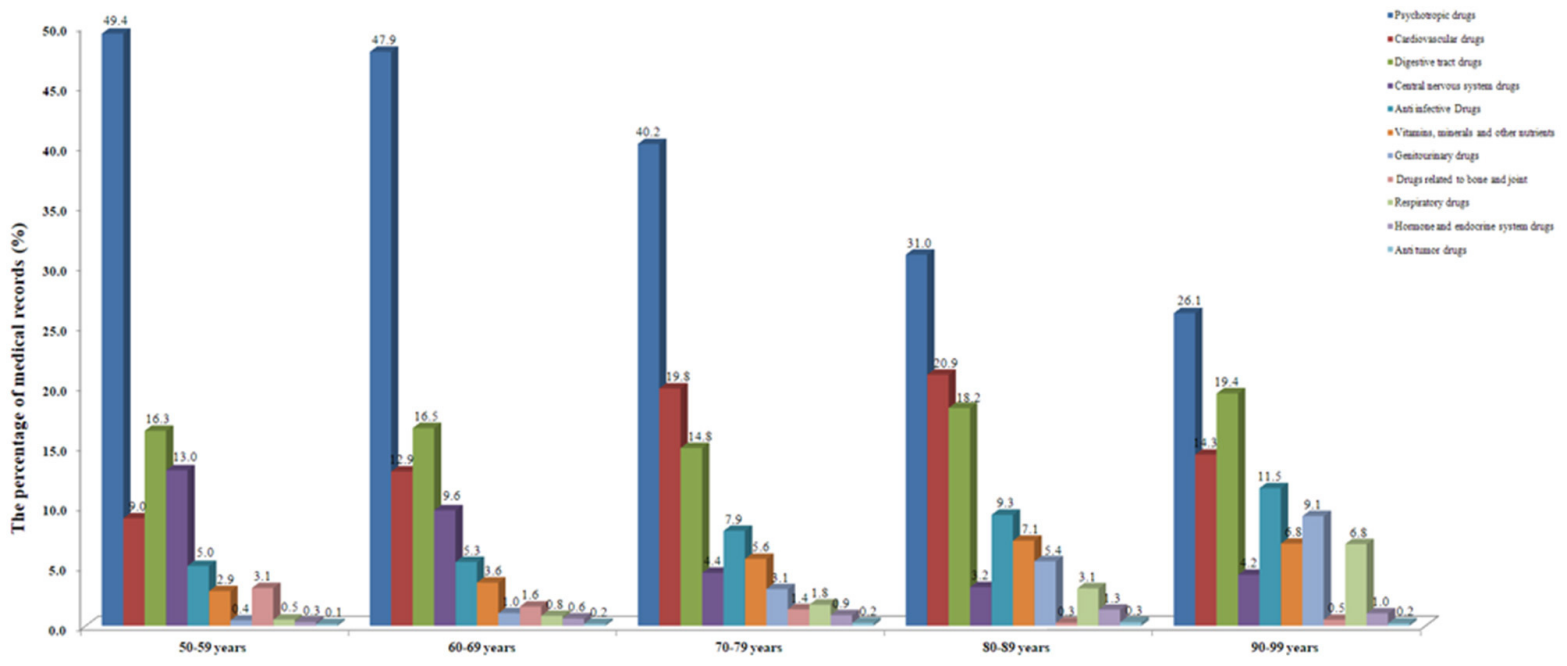
Age trend of drug prescriptions in the medical records in this study.

We then studied the changes in drug prescriptions and psychiatric diagnosis of older psychiatric patients according to age. Accordingly, we created five age groups: 50-59 years old, 60-69 years old, 70-79 years old, 80-89 years old, and 90-99 years old. The results showed that the number of older patients diagnosed by F00-F09 increased significantly with age. However, the number of F20-F29 patients decreased significantly with age. The percentages of other diseases diagnosed in older patients of different ages also varied greatly. For example, the highest percentages of F30-F39 patients were 60-69 years old (23.0%) and 70-79 years old (21.8%), but the percentages in 50-59 years old (13.7%), 80-89 years old (14.2%), and 90-99 years old (5.3%) were significantly reduced (Figure 3 and Supplementary Table 6).

**Figure 4:**
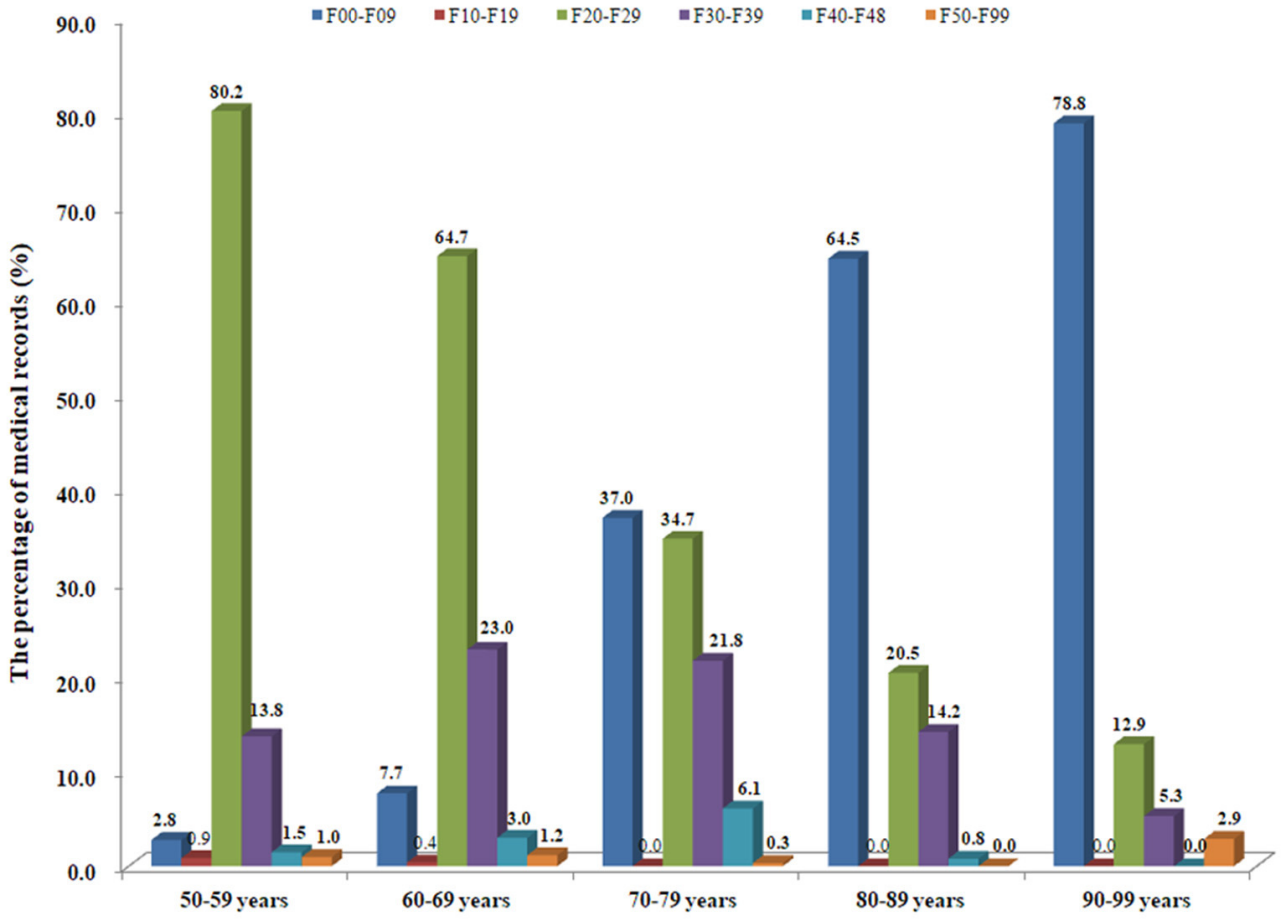
Age trend of major psychiatric diagnoses in the medical records in this study.

With regard to the age trend of drug prescription, the results showed that with increasing age, the prescription rate of antipsychotics decreased, while cardiovascular drugs, anti-infective drugs, urinary drugs, respiratory drugs and digestive tract drugs were exactly opposite. The percentage of prescriptions for other drugs also varied significantly in different age groups (Figure 4 and Supplementary Table 7).

## Discussion

In this retrospective, descriptive study, we examined the drug treatment of mental illness and physical comorbidities and explored the use of psychotropic drugs and physical disease drugs for different sexes and diagnosis. The age of female patients was significantly higher than that of male patients, which may be related to the longer life expectancy of Chinese women (33, 34) associated with the progress of medical technology, the higher possibility of treatment in today's Chinese society, and the higher survival age of older patients with mental illness. These data reflect that the number of older mental patients is gradually increasing in an aging society.

From our findings, mental and behavioral disorders due to psychoactive substance use (F10-F19) were more common in male patients than in female patients (*P* < 0.0001), consistent with their increased exposure to alcohol, drug, and other psychoactive substances (35, 36), whereas mood [affective] disorders were significantly more frequent in female than in male patients (*P* = 0.048), which is related to the higher incidence of depression in women (4). Correspondingly, there were sex differences in the prescriptions of antidepressant drugs (51.9% males vs. 45.4% females) and anti-anxiety drugs (male 36.9% vs. female 47%) (37), and depression accompanied by anxiety disorders or anxiety symptoms was more common in female patients (38, 39). In addition, there were also gender differences in the treatment of other physical comorbidities, such as the combination of antiarrhythmic drugs (male 19.5% vs. female 22.5%; *P* = 4.5208E-7) and anti-heart failure, anti-shock, and circulatory system drugs (male 30.8% vs. female 35.4%; *P* = 0.007), indicating that older female patients with mental illness were more likely to suffer from physical comorbidities, such as arrhythmia and heart failure.

However, compared with female patients, male patients used more mood stabilizers (male 18.5% vs. female 14.6%; *P* = 0.004) and genitourinary drugs (male 18.5% vs. female 14.6%; *P* = 0.004), while female patients used more endocrine system drugs (*P* = 0.006) (Table 2). These findings were consistent with previous results (26), showing that older male patients with mental illness were more likely to have somatic comorbidities in the genitourinary system, whereas female patients were more likely to have somatic comorbidities in the endocrine system due to hormonal changes. However, this difference may be particularly associated with hormones related to sex differences, which consequently lead to different metabolic disorder patterns (40). Epidemiological studies have showed that, compared with men, women have more sleep problems and depressive symptoms when sex hormones change (41), such as in adolescence, menopause, pregnancy and childbirth. In addition, premenstrual syndrome, premenstrual dysphoria, depression, and mood and anxiety disorders are found in a high proportion of women, which may be related to women's abnormal sensitivity to hormone fluctuations,. The influence of hormones on men may be more directly reflected in the urinary system. With aging, their urinary system is more prone to somatic diseases.

In this study, the number of patients in the first three ICD-10 diagnostic classifications (F20-F29, F30-F39, F00-F09) with the largest number of older mental patients accounted for 95.1% of the total in this study. Meanwhile, compared with that in 2005-2010, we found that the percentage of older patients with several major mental disease diagnoses increased, except F20-F29, from 2011 to 2015, especially diagnoses of F00-F09, F40-F48, and F30-F39 (Figure 1). Consistently, we found that the number of prescriptions for antidepressants and anti-Alzheimer's drugs more than doubled, and the number of prescriptions for anti-anxiety drugs and mood stabilizers increased by 80.6 and 50.0%, respectively, while antipsychotic drugs increased only 4.4% (Figure 2). This change may be related to China's social and economic development in recent years, increased social pressure, and a sharp increase in the prevalence of various major mental illnesses. It may also be related to changes in diagnostic criteria for mental illness and the level of clinical diagnosis and treatment of mental health in China.

Simultaneously, it is worth noting that the percentages of combined use of somatic disease treatment drugs varied for different diagnostic categories. For example, among the five primary diagnoses (Table 3) with the largest number of psychiatric hospitalizations, osteoporosis (7, 42), extravertebral system reaction (8, 43), dizziness and headache (44), and cardiovascular system disease (45) were the most common side effects of first- and second-generation antipsychotic drugs commonly used in clinical practice (46, 47). Therefore, compared with other diagnoses, patients with schizophrenia (F20) often used drugs related to bone and joint drugs (18.0%), cardiovascular drugs (74.3%), and central nervous system drugs (88.0%). The most commonly used central nervous system drugs were anti-tremor paralysis drugs (55.3%), which may be related to the side effects of first-generation antipsychotics (extrapyramidal side effects) (43, 48).

Because F01 patients were significantly older than the other patients in this study (Table 1), and because of the disease itself, these patients often experience prominent problems in cognitive function and sleep disorders, and often have physical dysfunctions, such as urinary incontinence and asthma. Therefore, patients with an F01 diagnosis were prescribed combinations of central nervous system drugs, such as sedative and hypnotic drugs (69.9%), and anti-Alzheimer's drugs (cognitive improvement drugs) (89.7%); cerebral metabolism and cerebral circulation improvement drugs (67.6%); and central nervous system agonists, dizziness drugs, and other central drugs (36.0%). In addition, they were prescribed drugs targeting the digestive system (97.8%), urinary system (67.6%), and respiratory system (59.6%), and almost all subcategories of cardiovascular drugs (97.1%) (Table 3, Supplementary Table 3). These data indicate that older F01 in-patients may suffer from a variety of physical diseases (≥2 types), suggesting that equal attention should be paid to treating physical diseases and mental conditions.

For F30-F39 patients, the combined use of antidepressants (72.6%) and mood stabilizers (57.3%) was the most common. This result suggested the credibility of the data in this study. In addition, F32 patients used the most anti-anxiety drugs (72.2%) (Table 3), as the prescription rate of anti-anxiety drugs in patients with depressive episodes (F32) was very high, suggesting the coexistence of anxiety and depression. This is consistent with reports stating that 37.3% of patients with depression had some type of anxiety disorder, while 74.6% of patients had depression and anxiety pain (49). Approximately 65.4 and 82.5% of older patients with F32 diagnosis used cardiovascular and central nervous system drugs (Table 3), suggesting that these patients also had cardiovascular and central nervous system-related diseases (11, 20, 50). For patients with F31 and F30 diagnoses, the combined use of digestive, cardiovascular, and nervous system drugs was very high, consistent with other studies (51).

Regarding drug use, the top three drugs with the highest prescription rate among older mental in-patients were psychotropic drugs (99.9%), central nervous system drugs (85.2%), and digestive system drugs (83.5%). In addition, 72.5% of older patients with mental illness received cardiovascular system drugs (circulatory system drugs) (Table 2), suggesting that the central nervous system, digestive system, and cardiovascular system-related diseases may be the most common physical complications in older patients with mental illness. However, unlike some previous reports (7, 8), our results showed that drugs targeting the urogenital system, bones and joints, respiratory system, hormone, and endocrine system were prescribed to 16.2, 14.7, 14.2, and 12.6% of the patients, respectively. This may be related to the study design, in which we only included hospitalized patients, or related to ethnic differences. It is worth noting that the three medical treatment drugs (central nervous system drugs, digestive system drugs, and cardiovascular system drugs) with the highest prescription rates are very widely used in older psychiatric in-patients, and the prescription rate only slightly lower than that of psychotropic drugs, suggesting that we should break through professional barriers between psychiatric specialist hospitals and general hospitals in the future. Chronic somatic comorbidities should be regarded as important in mental illnesses. Moving collaborative prescription and physical examination (22) into the forefront of psychiatric treatment and research (21), and their comprehensive application in clinical practice may be more conducive to the health maintenance and functional recovery of older psychiatric patients and improve their quality of life (42).

Finally, we studied the age trend of psychiatric diagnosis and the number of prescriptions for psychotropic drugs. The results showed that with increasing age, the percentage of older patients diagnosed with F20-F29 decreased, while F00-F09 increased (Figure 3). Consistently, the percentage of prescriptions for antipsychotics decreased, while cardiovascular drugs, digestive system drugs, urinary system drugs, respiratory system, anti-infective drugs, etc. increased (Figure 4). On the one hand, this finding once again proved that serious mental diseases such as schizophrenia affected life expectancy. On the other, it also showed that aging was a major risk factor for comorbid physical diseases of older patients with mental diseases, and further showed that older mental patients should be more prone to suffering from cardiovascular system, urinary system, digestive system, nervous system, respiratory system, and bone and joint system related diseases, which warned us that special attention should be paid to the treatment and prevention of somatic comorbid diseases in older psychiatric patients.

The strength of this study is that it specifically focused on older patients with mental illness and utilized a complete medical order database, which can more accurately reflect the prescription of all drug treatments for both mental diseases and physical complications. Furthermore, our data also explored the influence of different diagnoses, sex, and other factors on medical treatment of somatic comorbidities. However, there are some limitations in this study. First, we only considered the category of drugs, not the dose, course of treatment, and number of combined drugs; therefore, it was impossible to distinguish one type of somatic disease from multiple somatic diseases. Second, the discharge diagnosis information of some patients was missing, while the admission diagnosis information was complete in our medical record system, we thereby adopted the admission diagnosis for data analysis in this study. However, the discharge diagnosis information sometimes changes, which may limit the causal inference. Third, this study could only indirectly study the physical complications by virtue of treatment drugs, and lacked the accurate diagnosis of physical diseases assigned in general hospitals. Fourth, we are unable to obtain relevant data on patients' economic and social factors, medical insurance information, and living habits, such as smoking and drinking. As we did not analyze these factors, we cannot comment on their effects, which may limit the conclusions of this study. Fifth, this is only a retrospective study, which needs to be further studied and confirmed by prospective follow-up cohort studies in the future. Finally, it is impossible to distinguish the difference in treatment drugs needed by older patients with ≥1 physical diseases.

In summary, this study found that the combined drug treatment of somatic diseases, particularly for central nervous, digestive, cardiovascular, respiratory, genitourinary, and bone and joint problems, was extremely common among older psychiatric in-patients in China, and their percentages varied among the differing diagnoses of mental diseases. With increasing age, older patients with F20-F29 diagnosis decreased, while patients with F00-F09 increased; the prescription of antipsychotics decreased, and almost all drugs for comorbidity treatment increased. Compared with that in 2005-2010, older patients with other diagnosis, except F20-F29, increased between 2011 and 2015, as did the prescriptions for psychotropic drugs, genitourinary drugs, and cardiovascular drugs.

## Data Availability Statement

The original contributions presented in the study are included in the article/Supplementary Material, further inquiries can be directed to the corresponding author/s.

## Ethics Statement

The studies involving human participants were reviewed and approved by the Ethics Committee of Shanghai Mental Health Center. Written informed consent was not provided because this is a retrospective data analysis, and the data was extracted from the hospital medical record r system, which dated back to 2005.

## Author Contributions

HL and YZ conceived and designed the study. XWu, XWa, and PS assisted in data collection. KH and YY contributed to the statistical analysis. JR, JZ, and RM wrote the first draft of the paper. TY, LY, PJ, and XS commented significantly to the draft of the paper. CZ and YF finalized the manuscript. All authors have read and approved the final version of the manuscript.

## Funding

This work was supported by Grant from the Natural Science Foundation of China (81801338, 81771465, and 81930033), the National Key Research and Development Program of China (2016YFC1307100), the National Key Technologies R&D Program of China (2012BAI01B04), Shanghai Key Project of Science and Technology (2018SHZDZX05), Shanghai Mental Health Center Medical Youth Talents Flying Plan (2018-FX-03), the Sanming Project of Medicine in Shenzheng (SZSM201612006), and also supported by the Innovative Research Team of High-level Local Universities in Shanghai.

## Conflict of Interest

The authors declare that the research was conducted in the absence of any commercial or financial relationships that could be construed as a potential conflict of interest.

## Publisher's Note

All claims expressed in this article are solely those of the authors and do not necessarily represent those of their affiliated organizations, or those of the publisher, the editors and the reviewers. Any product that may be evaluated in this article, or claim that may be made by its manufacturer, is not guaranteed or endorsed by the publisher.
